# Efficient nucleic acid delivery to murine regulatory T cells by gold nanoparticle conjugates

**DOI:** 10.1038/srep28709

**Published:** 2016-07-06

**Authors:** Lisa Gamrad, Christoph Rehbock, Astrid M. Westendorf, Jan Buer, Stephan Barcikowski, Wiebke Hansen

**Affiliations:** 1 Technical Chemistry I and Center for Nanointegration Duisburg-Essen (CENIDE), University of Duisburg-Essen, Universitaetsstr. 7, 45141 Essen, Germany; 2 Institute of Medical Microbiology, University Hospital Essen, University Duisburg-Essen, Hufelandstr. 55, 45147 Essen, Germany

## Abstract

Immune responses have to be tightly controlled to guarantee maintenance of immunological tolerance and efficient clearance of pathogens and tumorigenic cells without induction of unspecific side effects. CD4^+^ CD25^+^ regulatory T cells (Tregs) play an important role in these processes due to their immunosuppressive function. Genetic modification of Tregs would be helpful to understand which molecules and pathways are involved in their function, but currently available methods are limited by time, costs or efficacy. Here, we made use of biofunctionalized gold nanoparticles as non-viral carriers to transport genetic information into murine Tregs. Confocal microscopy and transmission electron microscopy revealed an efficient uptake of the bioconjugates by Tregs. Most importantly, coupling eGFP-siRNA to those particles resulted in a dose and time dependent reduction of up to 50% of eGFP expression in Tregs isolated from Foxp3eGFP reporter mice. Thus, gold particles represent a suitable carrier for efficient import of nucleic acids into murine CD4^+^ CD25^+^ Tregs, superior to electroporation.

CD4^+^ CD25^+^ regulatory T cells (Tregs) play a crucial role in the control of autoimmunity and immune homeostasis. Based on their immunosuppressive function they represent attractive targets for the modulation of a broad variety of immune responses. However, for therapeutic application knowledge about their development and mode of action is indispensable. Over the last few years several molecules have been identified that were associated at least in part with the inhibitory activity of these T cell subset including CTLA-4, Lag3, Galectin-1, Neuropilin-1 and CD83^1^,^2^,^3^,^4^,^5^. In addition, the transcription factor Foxp3 has been described to be essential for their development and function^6^. Although these molecules have an impact on the phenotype and function of Tregs, the exact mode of action in particular during certain immune responses is not completely understood.

One common method to identify molecules and pathways involved in Treg function is the use of knock-out (KO) mice at least in the murine system. However, depletion of gene expression mostly affects the whole organism. To circumvent this problem, one might use conditional KO mice based on the Foxp3-cre (FIC) mouse line^1^, but this requires availability of respective floxed mice. As an alternative approach it would be helpful to down-regulate the gene of interest directly in the Tregs by transfection with siRNA molecules prior to detailed analysis of the impact on Treg phenotypic and functional properties. However, it is very difficult to genetically modify T cells. Viral-vector based transduction methods showed relative high efficacy in expressing genes of interest in non-regulatory T cells and hematopoetic cells^5^,^7^,^8^, but are limited by the time and costs for vector production in addition to safety issues. Optimized electroporation protocols in particular Lonza nucleofection yielded in up to 80% of viability and 40–60% of expression in human T cells^9^. However, in murine T cells, which are usually harder to transfect, applying these technique resulted in 35–55% viability and 20–40% expression of the gene of interest^10^. In addition, CD4^+^ T cells showed lower expression rates using RNA electroporation methodology in comparison to CD8^+^ T cells^11^. Thus, there is a clear need for the development of new cheap, easy to handle and efficient methods for genetic modulation of CD4^+^ T cells in particular murine Tregs.

For this purpose, gold nanoparticle based species may be a suitable tool. Gold nanoparticles are already widely used in biomedical applications like drug delivery^12^,^13^, nanotargeting and bioimaging^14^,^15^, amongst others due to their very good biocompatibility^16^. Additionally, these nanoparticles have a high affinity to thiolated biomolecules^17^ like oligonucleotides or peptides which makes it easy to create individual and functional species. A suitable way to generate nanobiconjugates with defined surface structures is pulsed laser ablation in liquids (PLAL). In contrast to chemical synthesis, this method yields totally ligand-free nanoparticles^18^,^19^ and hence does not require potentially toxic surfactants. Since laser ablation in water yields nanoparticles with broad size distributions, low salinity solutions of e.g. sodium phosphate buffer or sodium chloride can be used^20^ for size quenching and reduction of polydispersity. This allows the generation of monodisperse gold nanoparticles at particle sizes < 10 nm^21^,^22^. As laser-generated AuNPs possess totally a ligand-free surface, they can be easily functionalized with biomolecules reaching a five times higher surface coverage avoiding ligand-exchange^23^ and a controlled ligand load on the nanoparticle surface^20^,^21^,^23^. In this study, we chose ligand-free 5 nm gold nanoparticles as transporter platform which were functionalized by a combination of an oligonucleotide and a cell penetrating peptide. The latter one, a nuclear localization signaling sequence (NLS), was chosen to reach an efficient translocation of the transporter species^24^. Since the combination of positively charged peptides and negatively charged nanoparticles leads to charge compensation and therefore to the formation of agglomerated and presumably unstable conjugates^21^, oligonucleotides were used for pre-stabilization. These nanobioconjugates were examined for their potential to efficiently transfer siRNA into unstimulated murine CD4^+^ CD25^+^ Tregs.

## Results and Discussion

### Characteristics of gold nanoparticle-oligonucleotide-peptide conjugates

**Figure 1:**
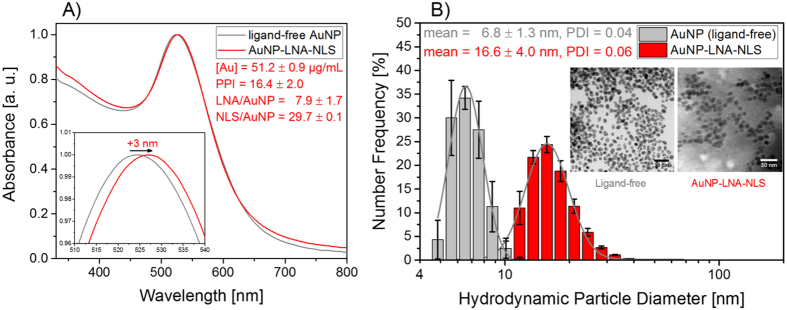
Properties of the gold nanoparticle conjugate. (**A**) Absorbance spectrum of gold nanoparticle-LNA-NLS-conjugates compared to ligand-free AuNPs; the inset depicts a zoom of the SPR-Peaks. (**B**) Number distribution of ligand-free AuNP and the AuNP-LNA-NLS-conjugate measured by DLS, the inset depicts a TEM-picture of the nanoparticle conjugates.

The aim of this study was to achieve cell penetration of primary, murine regulatory T cells by the utilization of gold nanoparticle bioconjugates and additionally, to show a successful functionality of these species inside the cells. For this aim, a special design of a biofunctional species was necessary where the functionality can be designed individually. Hence, nanoparticle conjugates were prepared by using laser generated, ligand-free gold nanoparticles with a primary particle diameter of 5.2 ± 0.7 nm as platform^21^. The number weighted hydrodynamic diameter (mean-value) determined by DLS is 6.8 ± 1.3 nm (Fig. 1B). These nanoparticles were functionalized with a NLS-peptide providing the conjugate the ability to cross the cell membrane and enter into the cell. The oligonucleotide utilized for pre-stabilization is a locked nucleic acid (LNA) which is a modified DNA-oligonucleotide. This molecule is more rigid than a DNA and hence, fewer molecules are necessary for nanoparticle stabilization leaving enough space on the particle surface for the peptide and a second functional molecule^24^. Both molecules were covalently bound to the nanoparticles via a thiol-bond in order to achieve a better stabilization and a defined system with well-known conditions. The improved stability due to the usage of a thiol-bond can be seen from the comparison with a non-thiol-bond which is depicted in the supporting information (Fig. S1). Figure (1A) shows an UV-Vis spectrum of the conjugates compared to ligand-free nanoparticles. The 3 nm shift of the surface plasmon resonance peak (SPR) indicates the successful functionalization of the gold nanoparticles by LNA and NLS. For a better illustration of deviating peak positions, the first derivative of the spectra was calculated, which is shown in Fig. S2. Here the root of the deviated function signifies the peak position. The gold nanoparticle-oligonulcotide-peptide conjugates that were used within these experiments carried 7.9 ± 1.7 LNA and 29.7 ± 0.1 NLS per AuNP which leads to stable bioconjugates with a gold concentration of 51.2 ± 0.9 μg/mL and a hydrodynamic diameter of 16.6 ± 4.0 nm (Fig. 1B). The bigger size of the conjugates compared to the primary particles is due to the ligand shell of oligonucleotide and peptide. The effect of stabilization using LNA can be seen in the Primary Particle Index (PPI), which gives a high value for low agglomeration and is determined by UV-Vis spectrometry^20^,^21^. Our system has a PPI of 16.4 ± 2.0 and a zeta potential of −48.1 ± 2.4 mV indicating a good colloidal stability. In case similar constructs were designed without LNA, the aggregation tendency was higher (PPI = 5), which can be derived from an increasing extinction in the NIR-region at e.g. 800 nm, verifying the necessity of further stabilization by nucleotides (Fig. 1, Suppl. Figs S3 and S4).

### Uptake of gold nanoparticle conjugates by murine splenocytes

First of all, we analyzed whether AuNP-LNA-NLS conjugates interact with different murine immune cells. For this purpose, we used an AF488 labeled oligonucleotide during particle conjugation and incubated the conjugates at different concentrations with splenocytes freshly isolated from wildtype BALB/c mice. After 4 h we determined the percentage of labeled AF488^+^ viable cells by flow cytometry.

**Figure 2:**
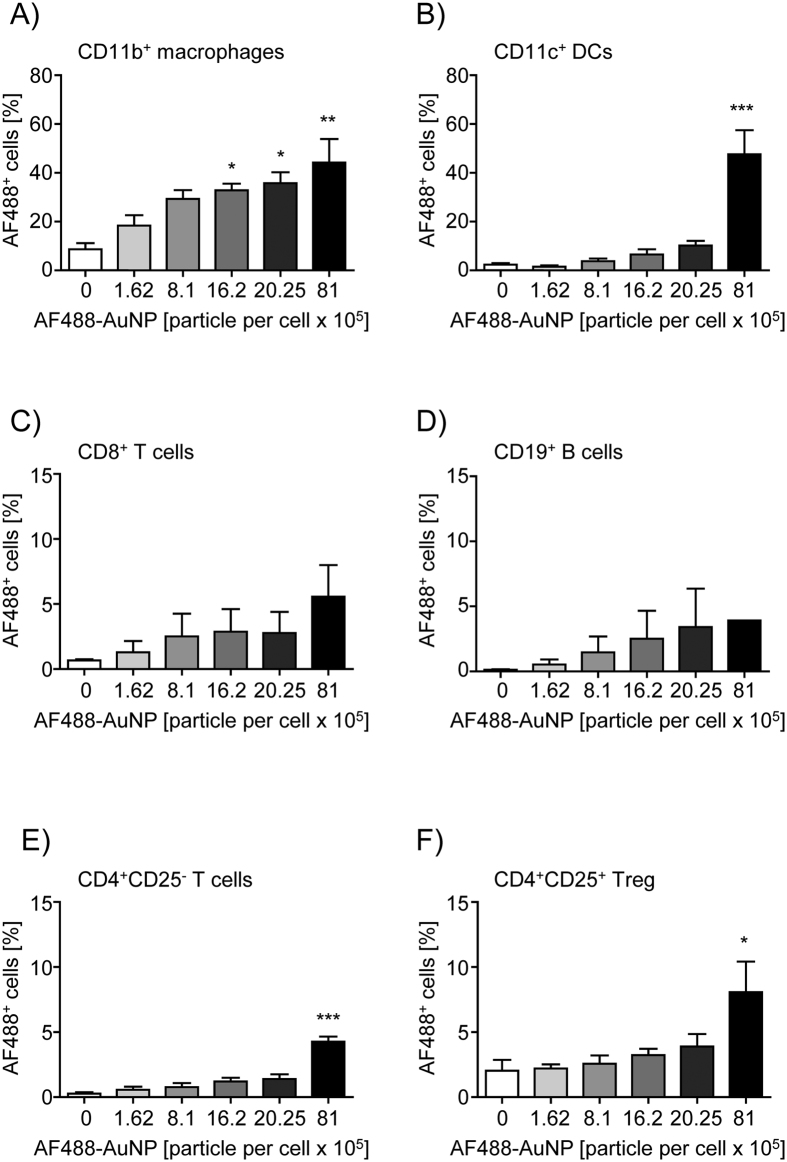
Uptake of the gold nanoparticle conjugates. Murine splenocytes were cultured in the absence or presence of 1.62 × 10^5^, 8.1 × 10^5^, 16.2 × 10^5^, 20.25 × 10^5^ or 81 × 10^5^ AF488-labeled nanoparticle conjugates (5 nm) per cell for 4 h. The amount of AF488 positive cells was analyzed on the respective living cell subpopulation (**A**) CD11b^+^ macrophages, (**B**) CD11c^+^ DCs, (**C**) CD8^+^ B cells, (**D**) CD19^+^ T cells, (**E**) CD4^+^ CD25^−^ T cells and (**F**) CD4^+^ CD25^+^ regulatory T cells by flow cytometry. Results from three independent experiments are shown as mean ± SEM. One-way ANOVA with Dunett’s post test was used for statistical analysis. *p < 0.05, **p < 0.01, ***p < 0.001.

As expected CD11b^+^ macrophages and CD11c^+^ dendritic cells (DCs) exhibited the highest capability of AuNP uptake in a dose dependent manner presumably due to their natural endocytotic activities (Fig. 2A,B, Suppl. Table S1). Indeed, efficient uptake of AuNPs by DCs and macrophages has been shown and was proposed to be related to pinocytosis or receptor-mediated endocytosis^25^,^26^. Approximately 3–5% of CD8^+^ T cells and CD19^+^ B cells showed an AF488 signal after incubation with the highest dose of AF488-conjugated particles (Fig. 2C,D, Suppl. Table S1). Interestingly, among CD4^+^ T cells, 8–10% of CD25–expressing Tregs were able to bind or take up the conjugates whereas only 5% of CD4^+^ CD25^−^ T cells showed AF488-derived fluorescence after treatment with 8.1 × 10^6^ fluorochrome-labeled particles (Fig. 2E,F, Suppl. Table S1). These results indicate that all immune cells analyzed were able to interact with AuNPs to a certain extent in a dose dependent manner.

However, by flow cytometric analysis we were not able to dissect whether the conjugates just bind to the surface of lymphocytes or whether they were taken up. Therefore, we performed confocal microscopy analysis of CD4^+^ T cells treated with AF488-labeled particle conjugates.

**Figure 3:**
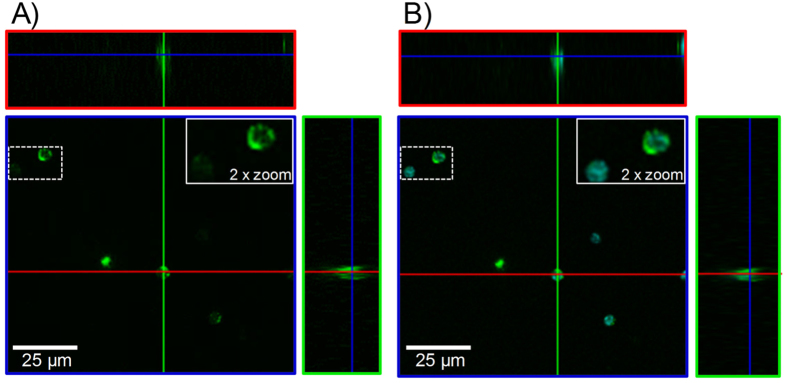
Confocal microscopy pictures of CD4^+^ CD25^+^ regulatory T cells incubated with gold nanoparticle conjugates. (**A**) Depiction of the nanoparticle conjugates (green) taken up by the T cells. (**B**) Depiction of nanoparticle conjugates (green) internalized by T cells with additional DAPI staining of the cells (cyan).

As depicted in Fig. 3 nanoparticle conjugates (green) were not only found to be accumulated on the surface of the cells (blue) but also inside the cells. The presented section of the confocal microscopy analysis shows that nanoparticles are spread all over the cell area. Nevertheless, there are some more intense spots on the inside of the cell membrane which may indicate a higher accumulation of nanoparticle conjugates outside the nucleus e.g. in vacuoles. Another evidence for the successful uptake of the nanoparticles is given by the view to the z-axis of the image. This furthermore demonstrates the intracellular distribution of nanoparticles over the whole cell area (Suppl. Fig. S5).

**Figure 4:**
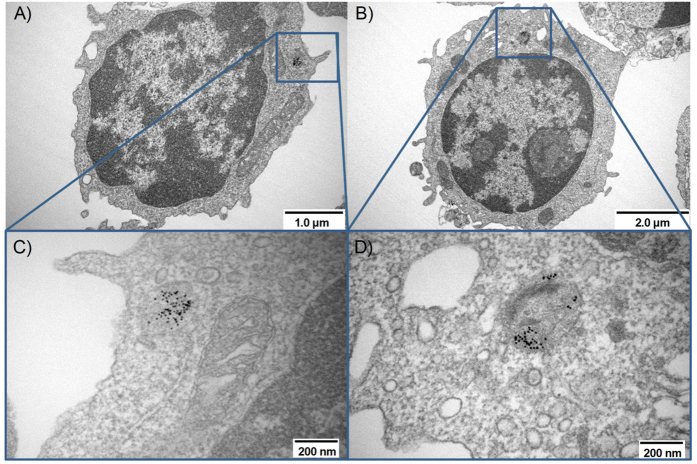
Uptaken nanoparticles found in vesicles inside the cytoplasm. (**A**,**B**) show an overview of the cells. (**C,D**) show a magnification of the internalized nanoparticles located in intracellular vesicles. These magnified images are rotated by less than 180° compared to the overview which is due to the operation mode of the microscope.

Confocal microscopy analysis provided evidence for intracellular localization of Au-NPs after incubation with freshly isolated murine CD4^+^ T cells. To further confirm this finding and to visualize the intracellular localization in more detail, we performed transmission electron microscopy (TEM) analysis of freshly isolated murine CD4^+^ T cells treated for 3 h with AuNPs. As depicted in Fig. 4 we detected accumulation of AuNPs in some intracellular vesicles of CD4^+^ T cells. Localization of gold particles in intracellular vesicles upon uptake was also described for murine DCs^25^. Since CD4^+^ T cells showed tentacle-like structures at their surface, we presume endocytosis of AuNPs by these cells, with regard to their small size most likely pinocytosis^27^, that end up in endosomal structures. However, further experiments are required to define the exact molecular mechanism involved in AuNP uptake by primary murine lymphocytes.

Most importantly, we clearly demonstrated the intracellular localization and thereby providing proof-of principle for an efficient uptake of AuNP conjugates by murine primary CD4^+^ T cells.

### Down-regulation of eGFP expression in murine eGFP^+^ regulatory T cells by administration of siRNA-coupled nanoparticles

**Figure 5:**
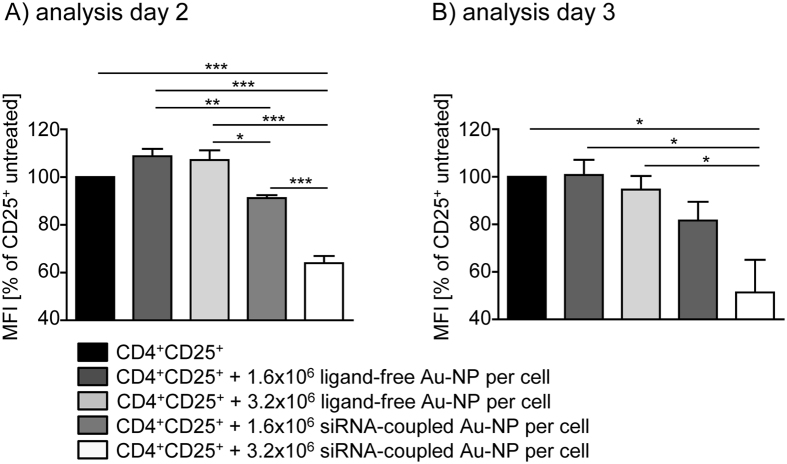
Down-regulation of eGFP-expression in eGFP^+^ murine regulatory T cells by siRNA-coupled gold particle conjugates. Freshly isolated eGFP^+^ regulatory T cells were cultured in presence or absence of 1.6 × 10^6^ or 3.2 × 10^6^ siRNA-coupled nanoparticle conjugates (5 nm) per cell or ligand-free particles as controls. eGFP expression was analyzed as mean fluorescence intensity (MFI) on gated living cells by flow cytometry (**A**) at day 2 or (**B**) day 3 and calculated as percentage of CD25^+^ untreated (= 100%). Results from three independent experiments are summarized as mean ± SEM. One-way ANOVA with Bonferroni´s post test was used for statistical analysis. *p < 0.05, **p < 0.01, ***p < 0.001.

To analyze whether one might utilize them as carriers for nucleic acids to modulate the gene expression profile of these cells, we additionally functionalized the gold nanoparticle conjugates via electrostatic interactions with siRNA directed against eGFP and incubated them with eGFP-expressing Tregs freshly isolated from Foxp3/eGFP-reporter mice. A dose of 1.6 × 10^6^ particles per cell was chosen for transfection experiments due to a successful uptake of AuNPs after incubation with this quantity of nanoparticles as demonstrated in Fig. 4. To further increase the efficiency of eGFP downregulation after AuNP-LNA-NLS + siRNA treatment, we have doubled the amount to 3.2 × 10^6^ particles per cell. Higher doses were not applied due to potentially toxic side effects. By this approach we were able to significantly down-regulate eGFP expression in a time and dose dependent manner. Incubation of murine Tregs with ligand-free nanoparticles did not alter the eGFP expression levels regardless of concentration and time point of analysis (Fig. 5, Suppl. Fig. S6, Suppl. Table S2).

Culturing eGFP^+^ Tregs with 1.6 × 10^6^ siRNA-coupled gold nanoparticles per cell resulted in a significant reduction of 8.7% in eGFP expression at day two which is further down-regulated (−18.3%) at day three (Fig. 5, Suppl. Table S2). Strikingly, treatment with 3.2 × 10^6^ AuNP-LNA-NLS + siRNA conjugates caused a reduction of 36% in eGFP expression at day two and a 48.8% lowered eGFP expression in viable murine Tregs at day three. While we detected uptake of AF488-labeled particles only in up to 10% of Tregs (Fig. 2, Suppl. Table S1), we achieved a much higher rate in down-regulation of eGFP expression after treatment with siRNA-coupled AuNPs. This discrepancy might be due to the longer incubation time (two or three days *vs.* four hours) and the use of purified eGFP^+^ Tregs in contrast to whole splenocytes used for our uptake experiments shown in Fig. 2. With these results and while we could not find nanoparticles inside the cytoplasm by TEM analysis, we assume that the physisorbed siRNA was released from the nanoparticles inside the vacuoles and therefore was able to penetrate them into the cytoplasm.

Successful transfection of T lymphocytes with plasmids or siRNA contructs was also achieved by others using transient plasmonic nanobubble injection^28^ or optimized electroporation protocols based on the Lonza nucleofection technique. Reduction in gene expression of 40–60% could be achieved for primary human lymphocytes, but only a very low percentage of primary murine T cells (10%) exhibited transgene expression upon electroporation^10^,^29^. By pre-activation of murine lymphocytes prior to electroporation transfection efficacies of up to 40% or 60% were achieved^10^,^11^. However, activation of T cells strongly modulates the molecular and functional properties of these cells and is therefore undesirable in many approaches. Thus, our results clearly demonstrate that AuNPs are efficient carriers for importing nucleic acids into un-stimulated murine regulatory T cells and that this method is superior to currently existing electroporation protocols.

We observed increasing numbers of dead cells after incubation of freshly isolated murine Tregs with siRNA-conjugated AuNPs in a time and dose dependent manner (Suppl. Fig. S7). Importantly, treatment of freshly isolated Tregs with AuNP-LNA-NLS (no siRNA) had no impact on cell survival, indicating that our AuNP conjugates themselves have no toxic side effects on murine T cells. The reason for the negative impact of eGFP-siRNA coupled to AuNPs on cell viability remains unclear, may be this is linked to the omittance of BSA^30^,^31^. However, siRNA transfer to unstimulated murine T cells by optimized electroporation protocol also resulted in a very low survival rate (15–28%)^11^. Thus, further optimization steps with regard to improve cell viability and efficacy in down-regulating gene expression by validating alternative siRNA-sequences have to be done in future studies. Nevertheless, our results provide proof-of principle for efficient nucleic acid delivery to murine Tregs by using small Au-NPs, providing a new technology to modulate the gene expression profile of these hard to transfect primary lymphocytes.

## Conclusions

In this study laser-based gold nanoparticle bioconjugates were shown to be a suitable tool for penetrating murine regulatory T cells and additionally can be used as efficient transporter for nucleic acids. This approach is a highly effective and very helpful method to modulate gene expression in primary Tregs with the aim to identify proteins and microRNAs that dictate the phenotype and function of these cells and thereby represents promising therapeutic targets. Our study provides proof-of principle for nucleic acid delivery into un-stimulated primary murine Tregs using gold nanoparticle bioconjugates with striking efficiencies. However, further engineering of bioconjugates in particular with regard to nucleic acid formulation is desirable to further reduce toxicity and further improve efficiency.

## Methods

### Preparation of gold nanoparticle conjugates

Ligand-free gold nanoparticles were prepared by laser ablation in liquid as described elsewhere^21^ using a picosecond laser system (10 ps, 160 μJ, 100 kHz, 16 W; Ekspla Atlantic). 600 μM sodium phosphate buffer at pH 8 was used as ablation liquid in order to achieve size quenched nanoparticles. While stirring the solution, the laser beam was moved in a spiral pattern on the target for 10 minutes achieving a bimodal size distribution. The nanoparticle generation was followed by a purification step removing particles >10 nm by ultracentrifugation (30,000 g, 13 minutes).

Ligand-free gold nanoparticles were subsequently conjugated *ex situ* by successive addition of the different ligands to the colloid. In the first step, oligonucleotide molecules (1.67 μM; HS-XYX XXY XXX YTT YXX XYX XYT X; X = 5′-Methyl-dC, Y = LNA thymine) were mixed with the colloid (50 μg/mL) and afterwards sodium chloride (0.5 M) was successively added slowly to the mixture to ease medium transfer. It was added in five steps in order to retain colloidal stability. After incubating the sample for 24 hours at room temperature, unbound ligands were removed by ultracentrifugation (100,000 g, 60 minutes). Finally, NLS-peptide (2 μM, CWG_3_PK_3_RKVED) was added to the redispersed nanoparticle-oligonucleotide conjugate followed by BSA (2.5 g/mL) for additional stabilization. BSA stabilization was only used during the experiments concerning nanoparticle uptake. For FACS and Confocal analysis the used oligonucleotide was labeled by AlexaFlour488 (AF488).

Regarding the down-regulation experiments, 40 μM siRNA (Sense: 5′ AGC UGA CCC UGA AGU UCA UTT 3′, Antisense: 3′ TTU CGA CUG GGA CUU CAA GUA 5′) was added after the peptide conjugation. For these experiments, BSA was not used as stabilizer in order to not impede the functionality of siRNA.

Nanoparticle conjugates (AuNP-LNA-NLS) were assessed concerning the conjugation efficiency and stability by UV-Vis measurements using an evolution 201 by Thermo Fisher Scientific. Size and Zeta Potential measurements based on dynamic light scattering were conducted using a Malvern Zetasizer Nano ZS.

### Mice

Foxp3/eGFP mice (BALB/c, Jackson Laboratories)^32^ and BALB/c mice (Harlan Laboratories) were bred in-house and maintained under specific pathogen-free conditions at the University Hospital Essen, University Duisburg-Essen. All animal experiments were performed in accordance with the guidelines of the German Animal Protection Law and approved by the North Rhine-Westphalian State Agency of Nature, Environment and Consumer Protection (LANUV), Germany.

### Preparation of murine splenocytes, CD4^+^ T cells and CD4^+^ CD25^+^ regulatory T cells

Splenocytes were obtained from whole organ by rinsing with erythrocyte lysis buffer and washing with phosphate-buffered saline (PBS) supplemented with 2% fetal calf serum (FCS) and 2 mM EDTA. CD4^+^ CD25^+^ regulatory T cells and CD4^+^ T cells were separated from splenocytes by using the CD4^+^ CD25^+^ Regulatory T Cell Isolation kit (Miltenyi Biotech, Bergisch Gladbach, Germany) or CD4^+^ T Cell Isolation kit (Miltenyi Biotech, Bergisch Gladbach, Germany), respectively, according to the manufacturers recommendations.

### Uptake of gold nanoparticle conjugates

1 × 10^6^ splenocytes were cultured in a total volume of 200 μL Iscove’s Modified Dulbecco’s Medium (IMDM) supplemented with 10% FCS, 25 μM β-mercaptoethanol and 100 U/ml penicillin/streptomycin in the absence or presence of 4 μL, 20 μL, 40 μL, 50 μL or 200 μL of AuNP-LNA-NLS (5 nm) at 37 °C and 5% CO_2_. Thus the nanoparticle dose was 0–8.1 × 10^6^ nanoparticles per cell corresponding to 318 μm^2^/cell. After 4 h splenocytes were washed twice with PBS supplemented with 2% FCS and 2 mM EDTA and stained with the fixable viability dye eFlour780 (eBioscience, Frankfurt, Germany) and fluorochrome-labeled anti-CD4, anti-CD25, anti-CD8, anti-CD19, anti-CD11c and anti-CD11b antibodies (all from BD Biosciences, Heidelberg, Germany) at 4 °C for 10 min. The frequency of AF488 positive cells was analyzed on the respective viable cell subpopulation by flow cytometry. FACS analysis was performed with LSRII and DIVA software (BD Biosciences Heidelberg, Germany).

Additionally, the uptake of nanoparticle conjugates was investigated optically by confocal microscopy. For this 1 × 10^6^ MACS-sorted CD4^+^ T cells were incubated with 40 μL ligand-free or 40 μL AuNP-LNA-NLS ( = 1.6 × 10^6^ nanoparticles per cell; surface dose: 63 μm^2^/cell) for 2 h in 400 μL IMDM supplemented with 10% FCS, 25 μM β-mercaptoethanol and 100 U/ml penicillin/streptomycin at 37 °C and 5% CO_2_. Subsequently, cells were washed with PBS, fixed with 2% paraformaldehyde for 15 min, washed twice with PBS and embedded in 20 μL Roti^®^Mount Fluor Care (Roth, Karlsruhe, Germany). For detection of fluorochrome labeled particles we used a Leica TCS SP8 epifluorescence confocal microscope equipped with a white light laser which is picosecond pulsed (78 MHz) and possesses 1.5 mW per line. Furthermore, a HC PL APO CS2 oil objective with a magnification of 40, a numerical aperture of 1.3 and a working distance of 0.24 mm was used. The Leica LAS AD 3 software was used for the measurements and data evaluation. Nanoparticle conjugates were tracked by exciting the AF488-dye bound to the oligonucleotide at 495 nm and recording the emission between 515 nm and 600 nm. The cell nucleus was stained by DAPI which was excited at 405 nm and recorded between 555 nm and 600 nm. Both species were analyzed sequentially in order to exclude cross effects from the other excitation wavelength. Furthermore, the samples were recorded using the differential interference contrast (DIC).

As a third method for the analysis of the nanoparticle uptake transmission electron microscopy was applied. For this 1 × 10^6^ MACS-sorted CD4^+^ T cells were incubated for4 hours in 400 μL IMDM supplemented with 10% FCS, 25 μM β-mercaptoethanol and 100 U/ml penicillin/streptomycin with 40 μL of AuNP-LNA-NLS conjugate (50 μg/mL) reaching a final dosage of 1.6 × 10^6^ nanoparticles per cell (= 63 μm^2^/cell). After that, cells were washed two times in PBS and subsequently fixed in glutaraldehyde at a final concentration of 2.5% for 1 hour, followed by a washing step using PBS. After that, the fixation in 1% osmium tetroxide and a stepwise dehydration in an ethanol solution from 30% to 100% including the infiltration of uranyl acetate followed. Finally, the sample was infiltrated with epon using Epon-propylenoxide mixtures of increasing amount of epon. After complete polymerization, the embedded sample was trimmed and ultrathin sections of 50–60 nm were cut on a microtome and collected on copper grids. The samples were poststained with 1% uranylacetate solution and 0.4% lead citrate. For the analysis with transmission electron microscopy a Hitachi S-4000 Electron microscope was used at a magnification of around 80,000 and an acceleration voltage of around 80 kV.

### Downregulation of eGFP expression in murine eGFP^+^ regulatory T cells

5 × 10^5^ CD4^+^ CD25^+^ regulatory T cells isolated from Foxp3/eGFP reporter mice^32^ were cultured in the absence or presence of 20 μL and 40 μL AuNPs or AuNP-LNA-NLS + siRNA conjugates (= 1.6 × 10^6^ and 3.2 × 10^6^ nanoparticle per cell; 63 μm^2^/cell and 126 μm^2^/cell) in a total volume of 200 μL IMDM supplemented with 500 U/ml recombinant IL-2 (eBiocience, Frankfurt, Germany) for two or three days, respectively. Cells were washed with PBS supplemented with 2% fetal calf serum and 2 mM EDTA, stained with the fixable viable dye eFlour 780 (eBioscience Frankfurt, Germany) at 4 °C for 10 min. and washed again. The frequency of eGFP-expressing viable cells was analyzed by flow cytometry. FACS analysis was performed with LSRII and DIVA software (BD Biosciences Heidelberg, Germany).

### Statistical analysis

Statistical analyses were performed with One-way ANOVA (Bonferroni, Dunett) as indicated with significance set at the levels of *p < 0.05, **p < 0.01, and ***p < 0.001. All analyses were calculated with Graph Pad Prism 5.0 Software (Graph Pad Software, La Jolla, CA).

## Additional Information

**How to cite this article**: Gamrad, L. *et al*. Efficient nucleic acid delivery to murine regulatory T cells by gold nanoparticle conjugates. *Sci. Rep.* **6**, 28709; doi: 10.1038/srep28709 (2016).

## Supplementary Material

Supplementary Information
