# Supplementary material for: Efficient nucleic acid delivery to murine regulatory T cells by gold nanoparticle conjugates
Source: Sci Rep. 2016 Jul 6;6:28709. doi: 10.1038/srep28709 (PMC4933883; doi:10.1038/srep28709)
Supplement: Supplementary Information [file srep28709-s1.pdf]

Supplementary Information

Efficient nucleic acid delivery to murine regulatory T cells by gold nanoparticle conjugates

Gamrad, Lisa; Rehbock, Christoph; Westendorf, Astrid M.; Buer, Jan; Barcikowski, Stephan and Hansen, Wiebke

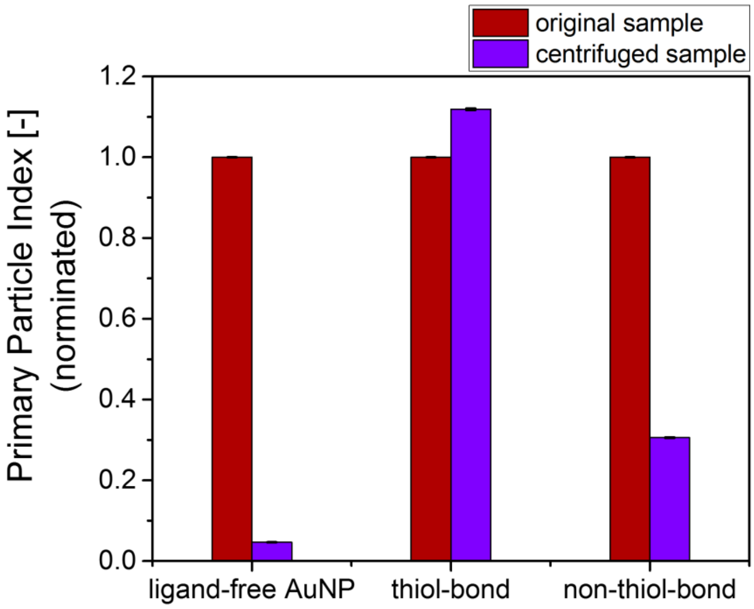

Figure S1: **Primary Particle Index of ligand-free gold nanoparticles and gold nanoparticle conjugates which were prepared with a thiolated and a non-thiolated peptide ligand.** Original and centrifuged samples were compared.

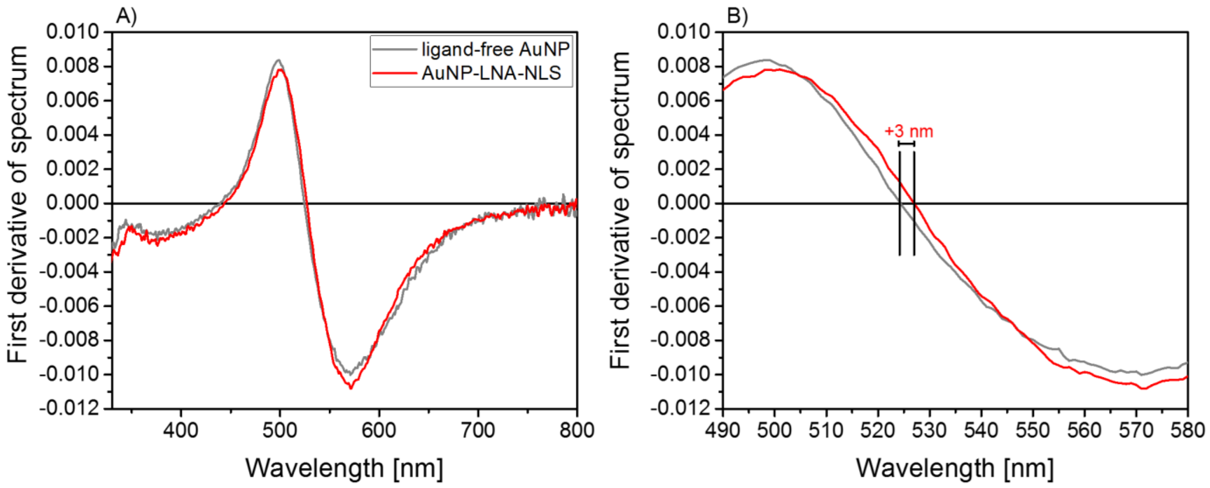

Figure S2: First derivative of the UV-vis spectra (A) and magnification of the First derivative of the UV-vis spectra (B) of ligand-free gold nanoparticles and AuNP-LNA-NLS conjugates.

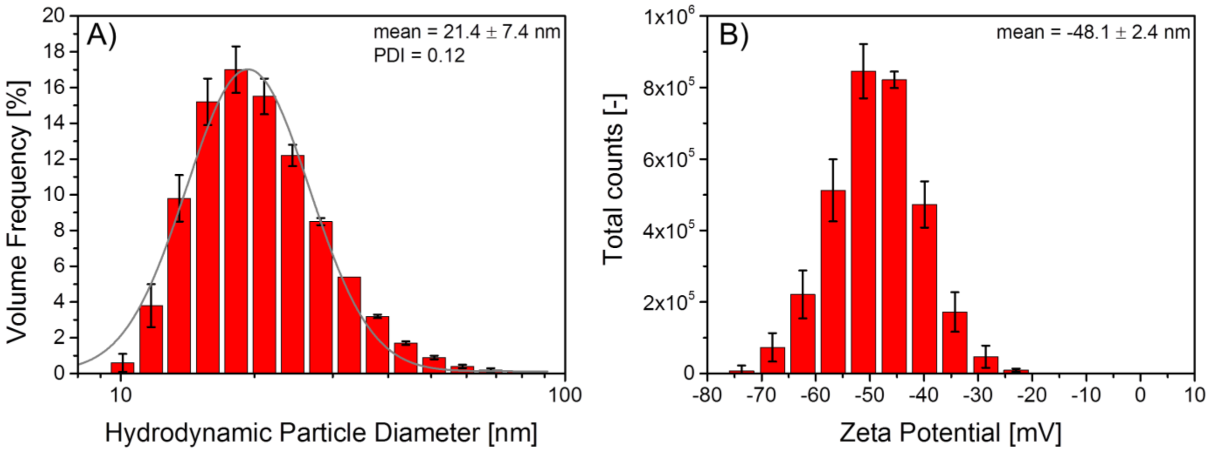

Figure S3: Characterization gold nanoparticle conjugates: A) Volume Distribution of gold nanoparticle conjugates measured by DLS. B) Distribution of Zeta Potential measured by DLS.

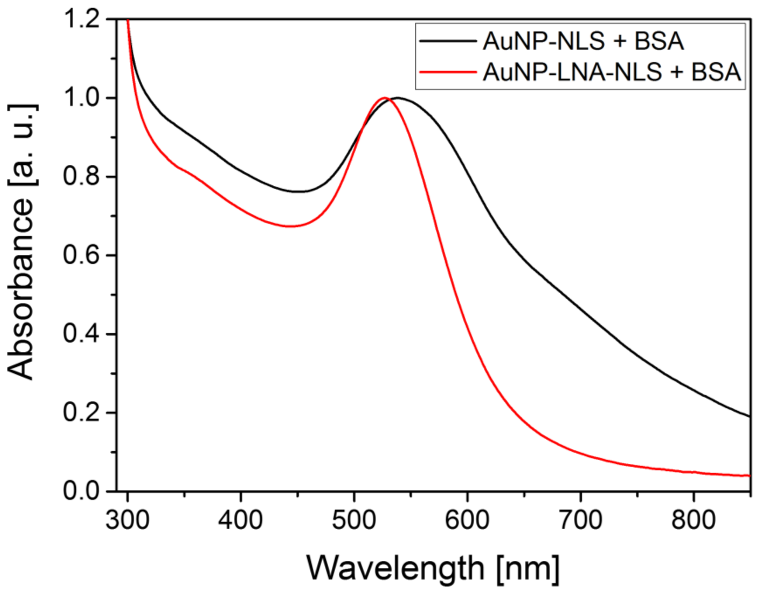

Figure S4: Absorbance spectra of AuNP-NLS + BSA and AuNP-LNA-NLS + BSA showing the broadening of the surface plasmon resonance peak indicating the agglomeration without using LNA for stabilization.

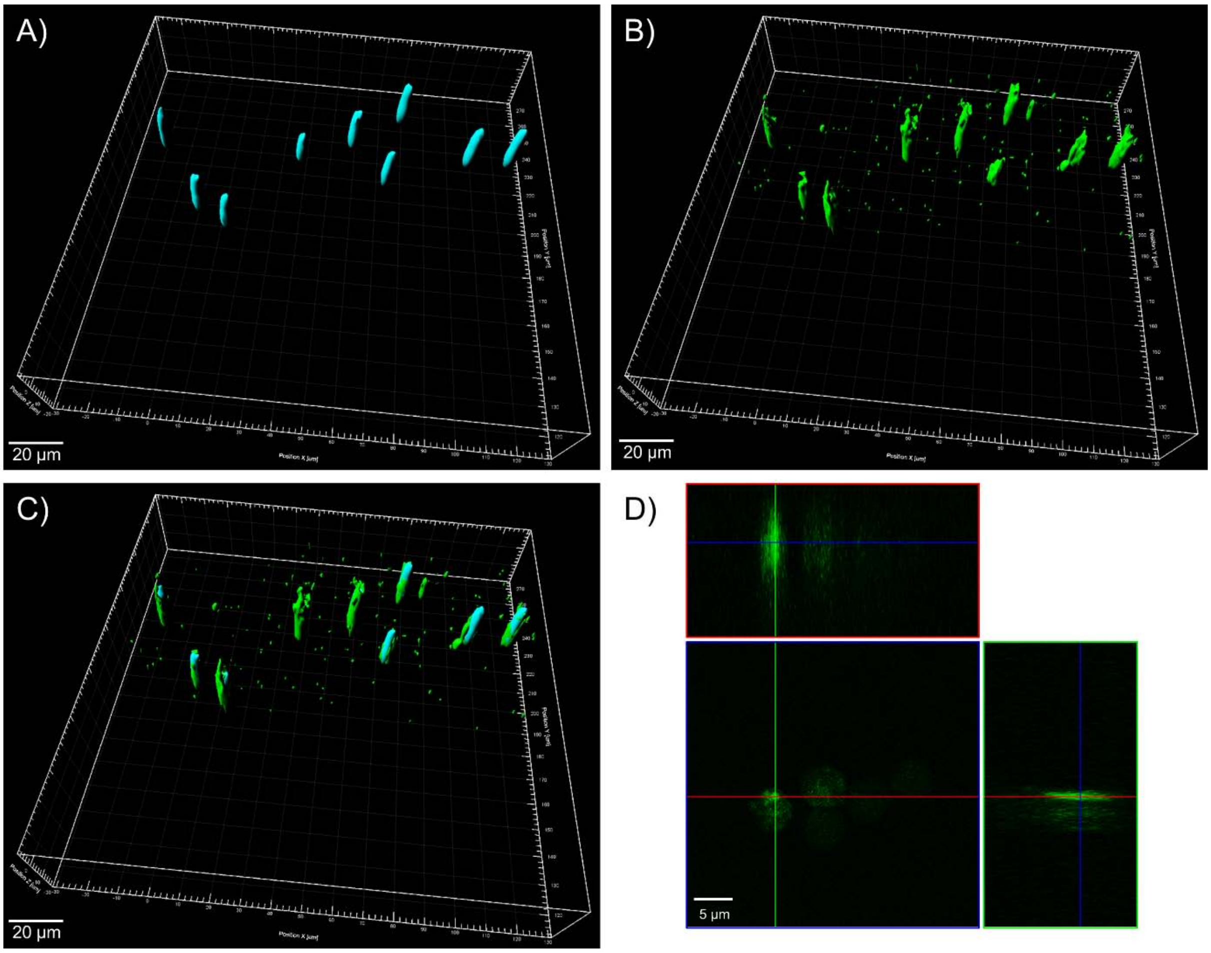

Figure S5: Confocal microscopy pictures of gold nanoparticle conjugates uptaken by T cells. 3D pictures depicting A) the T cells in cyan, B) the nanoparticles in green and C) an overlay of cells and nanoparticles. Confocal pictures were edited to improve the distinction of cells, nanoparticles and the background. D) confocal microscopy picture of one plane showing the nanoparticles (green) inside the cells.

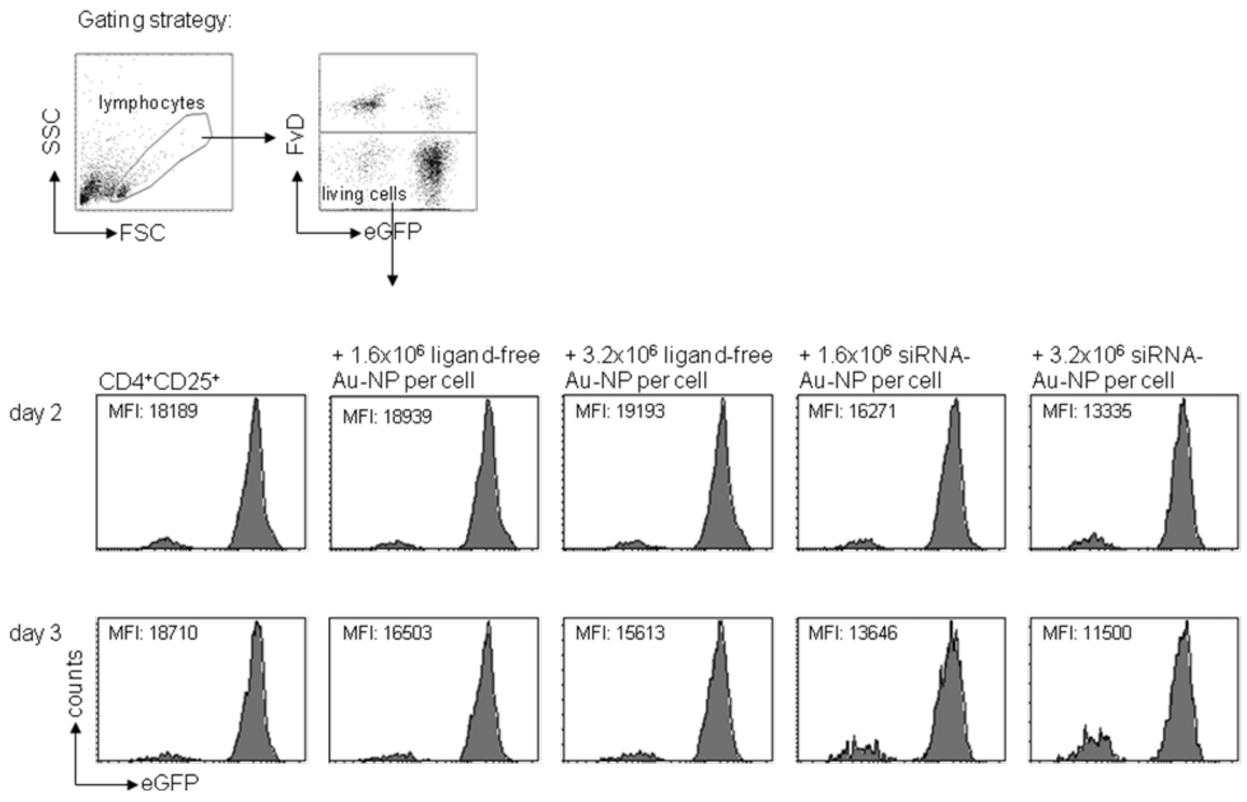

Figure S6: Gating strategy and representative histograms for analysis of eGFP-expressing CD4$^+$CD25$^+$ Tregs by flow cytometry. CD4$^+$CD25$^+$ Tregs were isolated from Foxp3/eGFP reporter mice, left untreated or incubated with indicated doses of ligand-free AuNP or siRNA-coupled AuNP for two or three days prior to analysis of eGFP expression as MFI on gated living cells by flow cytometry.

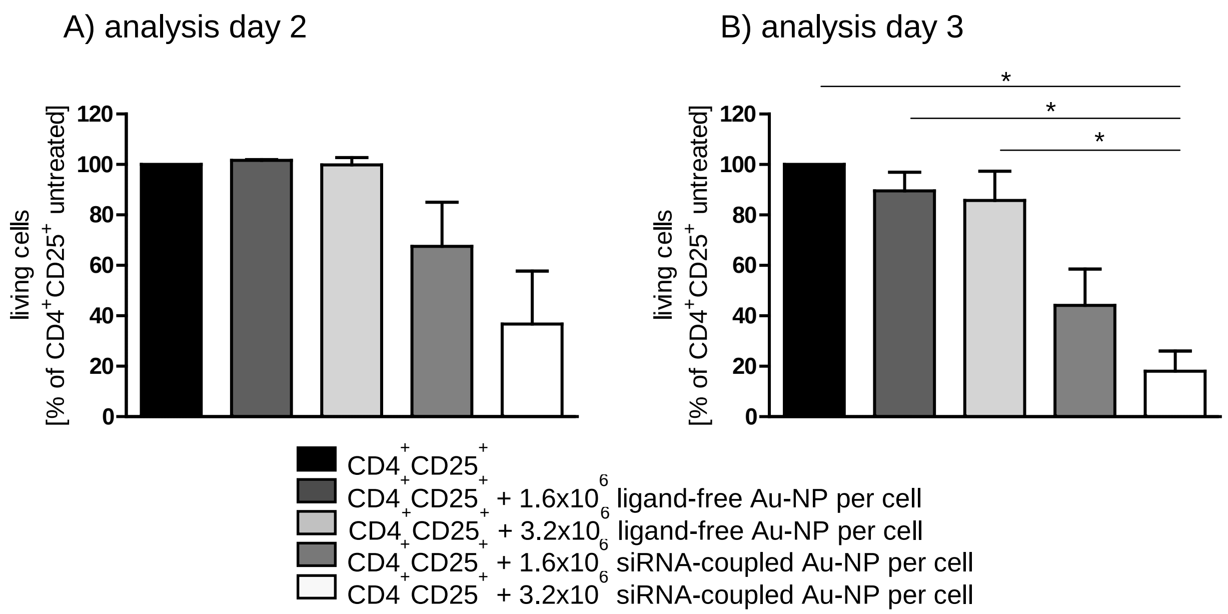

Figure S7: Survival rate of siRNA-coupled AuNP treated $CD4^{+}CD25^{+}$ regulatory T cells. Freshly isolated $eGFP^{+}$ regulatory T cells were cultured in presence or absence of $1.6x10^{6}$ or $3.2x10^{6}$ siRNA-coupled nanoparticle conjugates (5 nm) per cell or ligand-free particles as controls for A) two days or B) three days. Cells were harvested and stained with the fixable viability dye eFlour780. Percentages of living cells were determined by flow cytometry and calculated as percentage of untreated cells. Results from three independent experiments are summarized as mean ± SEM. One-way ANOVA with Bonferroni´s post test was used for statistical analysis. *$p<0.05$.

Table S1: Percentage of AF488^{+} cells after treatment with AF488-labeled AuNP for 4h.

	Treatment with AF488-labeled AuNP conjugates per cell					
	$0x10^{5}$ AF488-labeled AuNP	$1.62x10^{5}$ AF488-labeled AuNP	$8.1x10^{5}$ AF488-labeled AuNP	$16.2x10^{5}$ AF488-labeled AuNP	$20.25x10^{5}$ AF488-labeled AuNP	$81x10^{5}$ AF488-labeled AuNP
% AF488^{+} **CD11b^{+}** cells (mean)	8.667	18.400	29.300	32.830	35.770	44.130
Std error of mean	2.508	4.258	3.592	2.727	4.486	9.668
% AF488^{+} **CD11c^{+}** cells (mean)	2.367	1.533	3.767	6.500	10.200	47.550
Std error of mean	0.623	0.491	1.040	2.139	1.893	9.850
% AF488^{+} **CD8^{+}** cells (mean)	0.667	1.300	2.500	2.867	3.750	5.567
Std error of mean	0.088	0.862	1.756	1.736	2.250	2.431
% AF488^{+} **CD19^{+}** cells (mean)	0.133	0.533	1.467	2.500	3.400	3.900
Std error of mean	0.033	0.384	1.217	2.150	2.950	0.000
% AF488^{+} **CD4^{+}CD25^{-}** cells (mean)	0.267	0.567	0.767	1.200	1.400	4.267
Std error of mean	0.120	0.240	0.318	0.289	0.361	0.384
% AF488^{+} **CD4^{+}CD25^{+}** cells (mean)	2.033	2.200	2.567	3.233	3.900	8.067
Std error of mean	0.841	0.322	0.636	0.491	0.954	2.327

Table S2: eGFP expression of siRNA-coupled AuNP and ligand-free AuNP treated $CD4^+CD25^+$ regulatory T cells isolated from Foxp3eGFP reporter mice.

Cells	treatment	treatment duration	MFI [% of CD25+ untreated]	Std. error of mean
$CD4^+CD25^+$ T cells	no treatment	2 days	100.00	0.000
$CD4^+CD25^+$ T cells	$1.6x10^6$ ligand-free AuNP per cell	2 days	108.80	3.038
$CD4^+CD25^+$ T cells	$3.2x10^6$ ligand-free AuNP per cell	2 days	107.20	4.056
$CD4^+CD25^+$ T cells	$1.6x10^6$ siRNA-coupled AuNP per cell	2 days	91.26	1.172
$CD4^+CD25^+$ T cells	$3.2x10^6$ siRNA-coupled AuNP per cell	2 days	63.96	3.038
$CD4^+CD25^+$ T cells	no treatment	3 days	100.00	0.000
$CD4^+CD25^+$ T cells	$1.6x10^6$ ligand-free AuNP per cell	3 days	100.80	6.338
$CD4^+CD25^+$ T cells	$3.2x10^6$ ligand-free AuNP per cell	3 days	94.61	5.738
$CD4^+CD25^+$ T cells	$1.6x10^6$ siRNA-coupled AuNP per cell	3 days	81.66	7.809
$CD4^+CD25^+$ T cells	$3.2x10^6$ siRNA-coupled AuNP per cell	3 days	51.39	13.710
